# Effects of psychiatric history on cognitive performance in old-age depression

**DOI:** 10.3389/fpsyg.2015.00865

**Published:** 2015-06-29

**Authors:** Alexandra Pantzar, Anna Rita Atti, Lars Bäckman, Erika J. Laukka

**Affiliations:** ^1^Aging Research Center, Karolinska Institutet and Stockholm UniversityStockholm, Sweden; ^2^Bologna UniversityBologna, Italy; ^3^Stockholm Gerontology Research CenterStockholm, Sweden

**Keywords:** depression, remission, psychiatric history, old-age, cognition

## Abstract

Cognitive deficits in old-age depression vary as a function of multiple factors; one rarely examined factor is long-term psychiatric history. We investigated effects of psychiatric history on cognitive performance in old-age depression and in remitted persons. In the population-based Swedish National Study on Aging and Care in Kungsholmen study, older persons (≥60 years) without dementia were tested with a cognitive battery and matched to the Swedish National Inpatient Register (starting 1969). Participants were grouped according to current depression status and psychiatric history and compared to healthy controls (*n* = 96). Group differences were observed for processing speed, attention, executive functions, and verbal fluency. Persons with depression and psychiatric inpatient history (*n* = 20) and late-onset depression (*n* = 49) performed at the lowest levels, whereas cognitive performance in persons with self-reported recurrent unipolar depression (*n* = 52) was intermediate. Remitted persons with inpatient history of unipolar depression (*n* = 38) exhibited no cognitive deficits. Heart disease burden, physical inactivity, and cumulative inpatient days modulated the observed group differences in cognitive performance. Among currently depressed persons, those with inpatient history, and late onset performed at the lowest levels. Importantly, remitted persons showed no cognitive deficits, possibly reflecting the extended time since the last admission (*m* = 15.6 years). Thus, the present data suggest that cognitive deficits in unipolar depression may be more state- than trait-related. Information on profiles of cognitive performance, psychiatric history, and health behaviors may be useful in tailoring individualized treatment.

## Introduction

Depression is not only highly prevalent, but also a highly recurring disorder that causes emotional suffering along with reduced capacity to function (World Health Organization [WHO], 1993; APA, 2014).

Cognitive symptoms in depression are associated with longer episode duration, higher distress, reduced remission rates, and higher relapse rates (Dunkin et al., 2000; McCall and Dunn, 2003; Jaeger et al., 2006; Papakostas, 2014). However, reports on cognitive performance in old-age depression have yielded discrepant results, ranging from deficits in a large number of cognitive domains (processing speed; Butters et al., 2004; Köhler et al., 2010 attention; Ganguli et al., 2009; Thomas et al., 2009, executive functions; Beats et al., 1996; Lockwood et al., 2002, episodic memory; Bäckman and Forsell, 1994; Bäckman et al., 1996; Kramer-Ginsberg et al., 1999, semantic memory; Zakzanis et al., 1998; Herrmann et al., 2007, and spatial ability; Lesser et al., 1996; Elderkin-Thompson et al., 2004), to lack of any such deficits (Baune et al., 2007; Fischer et al., 2008; Krogh et al., 2012).

At least two types of old-age depression can be discerned. Early onset age (≤60 years) of recurrent depression, persisting into late life, and late onset age (>60 years) of the first depressive episode. The latter type has been suggested to be associated with vascular disease, and accompanied by pronounced executive dysfunctions (Alexopoulos, 1997, 2006). Besides age of onset, the neuropsychological profile of depression varies as a function of numerous other factors, including inpatient status, and cumulative illness burden (Lee et al., 2012; Hasselbalch et al., 2013).

Although cognitive performance for some domains may improve during remission of depressive symptoms (Biringer et al., 2005, 2007; Story et al., 2008; Hammar and Årdal, 2012; Lahr et al., 2014) most studies demonstrate incomplete cognitive recovery (Beats et al., 1996; Nebes et al., 2000; Portella et al., 2003; Yuan et al., 2008; Hasselbalch et al., 2011). Heterogeneity of cognitive deficits during acute versus remitted states has raised the question of whether these deficits are state- (present during acute states) or trait- (present both in acute and remitted states) related. However, a limitation of previous studies has been that participants have been followed for relatively short periods of time, which may account for the lack of cognitive recovery.

Investigation of the effects of long-term psychiatric inpatient history on depression-related cognitive deficits is important to deepen the knowledge regarding when cognitive deficits occur in depression. In the present project, we had access to inpatient psychiatric history spanning over 30 years. The chief objective was to study effects of psychiatric history on cognitive performance in old-age depression.

## Materials and Methods

### Participants

The Swedish National Study on Aging and Care in Kungsholmen (SNAC-K) is a longitudinal population-based study, including randomly selected older (≥60 years) persons. In total, 3353 persons completed baseline assessments consisting of a nurse interview, a medical examination and self-report questionnaires. Out of these, 2848 persons completed neuropsychological testing (see Laukka et al., 2013). SNAC-K has been approved by the ethical committee at the Karolinska Institutet, and the ethical guidelines from the Declaration of Helsinki are followed. Written informed consent was collected from all participants, or, if the person was severely cognitively impaired, from next-of-kin.

### Depression Diagnosis

Depressive symptoms were assessed with the Comprehensive Psychopathological Rating Scale (CPRS; Åsberg et al., 1978). Information relevant for depression diagnosis was also obtained from self-report questionnaires, thyroid function assessment, and the examining physician’s clinical judgment. A geriatric psychiatrist diagnosed unipolar depression according to ICD-10 criteria (World Health Organization [WHO], 1993). For a detailed description see Pantzar et al. (2014). In case of disagreement between different sources of information, a senior geriatric psychiatrist was consulted to make the final diagnosis. The psychiatrists were blind to pharmacological therapy, as well as to medical and psychiatric history.

### The Swedish National Inpatient Register

Swedish physicians are required to report to the National Inpatient Register (IPR). The register became national in 1969 and has been validated for research purposes (Ludvigsson et al., 2011). After matching SNAC-K participants to the IPR with personal identity numbers, information on primary psychiatric diagnoses was obtained from 1969 to baseline examination (2001–2004), thus covering up to 35 years. The ICD-8 and -9 primary psychiatric diagnoses were converted into ICD-10 categories. All persons with F00–F09 diagnoses were excluded. All data in this study, except the primary psychiatric diagnoses of admissions from the IPR, derive from the SNAC-K study.

### Dementia Diagnosis

Diagnosis of dementia was made according to DSM-IV criteria (APA, 2000) using a three-step procedure. First a preliminary diagnosis was made by the examining physician. Next, the preliminary diagnosis was compared to a second, independent, diagnosis made by another physician based on computerized data. In case of disagreement, a third physician was consulted to make the final diagnosis. Participants who were non-demented at baseline, but diagnosed with dementia during the follow-up period (maximum 6 years) were considered to be in a prodromal dementia phase at baseline (**Table 1**).

**Table 1:** Background, health, and clinical characteristics across depression status and psychiatric history.

	Healthy controls (HC) *n* = 96		Depressed no history (DNH), *n* = 49		Depressed self-reported unipolar depression (DSRH), *n* = 52		Depressed inpatient history mixed (DIPH), *n* = 20		Remitted inpatient history unipolar depression (RIPH), *n* = 38		*p*	η^2^/ø
Women: %	58.3		65.3		76.9		55.0		60.5		0.20	0.15
Age, baseline: M (SD)	71.3	(9.9)	80.3	(8.2)	74.5	(10.9)	71.8	(9.8)	68.3	(7.9)	0.00∗∗	0.15
Years of education: M (SD)	11.7	(4.0)	10.6	(3.8)	10.6	(3.8)	11.1	(4.6)	12.0	(3.9)	0.27	0.02
Age, first admission: M (SD)	–	–	–	–	–	–	55.9	(17.6)	51.9	(11.8)	0.31	0.02
Age, last admission: M (SD)	–	–	–	–	–	–	63.0	(14.8)	52.6	(12.5)	0.00∗	0.13
Psychiatric period, years: M (SD)	–	–	–	–	–	–	7.1	(8.8)	0.7	(2.1)	0.00∗∗	0.24
Years since last admission: M (SD)	–	–	–	–	–	–	8.9	(9.5)	15.6	(9.2)	0.01∗∗	0.11
No of admissions: M (SD)	–	–	–	–	–	–	5.2	(4.9)	1.7	(1.7)	0.00∗∗	0.22
No of inpatient days: M (SD)	–	–	–	–	–	–	175.3	(195.2)	41.1	(67.9)	0.00∗∗	0.21
MADRS: M (SD)	2.2	(3.7)	12.7	(4.6)	14.6	(4.8)	17.6	(6.7)	2.6	(3.2)	0.00∗∗	0.67
MMSE: M (SD)	29.1	(1.3)	27.6	(2.2)	28.5	(1.7)	27.9	(2.5)	28.8	(1.5)	0.00∗∗	0.10
Prodromal dementia: %		0		30.6		15.4		0		5.3	0.00∗∗	0.39
Heart disease burden: M (SD)	0.30	(0.65)	0.90	(0.98)	0.52	(0.83)	0.60	(1.05)	0.21	(0.47)	0.00∗∗	0.89
Vascular risk burden: M (SD)	0.97	(0.83)	1.25	(0.72)	0.94	(0.89)	1.20	(0.89)	1.03	(0.99)	0.31	0.02
Cerebralvascular disease: %		5.2		12.2		9.6		15.0		5.3	0.41	0.13
Physical inactivity: %		21.9		44.9		46.2		80.0		26.3	0.00∗∗	0.35
Any medication: %		0		6.1		32.7		65.0		39.5	0.00∗∗	0.53
TCAs: %		0		0		1.9		10.0		2.6	–	–
SSRIs: %		0		0		21.2		20.0		23.7	–	–
Other ADs: %		0		0		7.7		10.0		5.3	–	–
Lithium: %		0		0		0		10.0		2.6	–	–
Antipsychotics: %		0		6.1		3.8		40.0		5.3	–	–

*M, mean; SD, standard deviation; psychiatric period, age at last admission minus age first admission; MADRS, Montgomery and Åsberg Depression Rating Scale; MMSE, Mini Mental State Examination; prodromal dementia, persons receiving a DSM-IV dementia diagnosis at follow up 3 or 6 years after baseline examination; Heart disease burden: heart failure, coronary heart, atrial fibrillation; Vascular risk burden: hypertension stage 2, obesity, diabetes, current smoking, high cholesterol; Physical inactivity: never physically active or <2–3 times/month; TCA, Tricyclic antidepressant; SSRI, Selective serotonin reuptake inhibitors; Other antidepressants, Mirtazapin, Venlafaxin; ∗∗p < 0.01, ∗p < 0.05*.

### Study Sample

SNAC-K participants without dementia who completed neuropsychological testing during baseline formed the basis for the study sample. A group of healthy controls (HC, *n* = 96) was randomly selected and screened for depression, psychiatric inpatient history, stroke, cancer, Parkinson’s disease, diabetes, and medication that may have negative effects on cognitive performance (e.g., psychotropics, opioids, antiepileptics, glucocorticoids, anticholinergics).

Based on psychiatric history and current depression status (SNAC-K diagnosis), four groups were identified. The four groups were (1) late-onset, unipolar depression, no history (Depressed, No History; DNH, *n* = 49; mild: 57.1%, moderate: 38.8%, severe: 4.1%); (2) recurrent unipolar depression, self-reported history (Depressed, Self-Reported History; DSRH, *n* = 52; mild: 50.0%, moderate: 46.2%, severe: 3.8%); (3) depressed with a recurrent mix of psychiatric disorders, inpatient history [Depressed, InPatient History; DIPH, *n* = 20; mild: 45.0%, moderate: 35.0%, severe: 20.0%]; and (4) remitted unipolar depression, inpatient history [Remitted, InPatient History, RIPH, *n* = 38].

The group with a mixed psychiatric inpatient history had, in total, 114 admissions across five ICD-10 categories (Psychoactive substance use, F10–F19: *f* = 30; Schizophrenia and delusions, F20–F29: *f* = 18; Mood/affective disorders, F30–F39: *f* = 46; Neurotic, stress related, F40–F49: *f* = 19; and personality disorders, F60–F69: *f* = 1). Occurrence of multiple psychiatric categories was 45% (two categories: 30.0%; three categories: 15%). Within F30–F39, four persons had admissions for bipolar disorders (15 admissions). The remitted group with an inpatient history had, in total, 67 admissions for unipolar depression only.

### Neuropsychological Test Battery

*Short-term memory* was assessed by Digit Span (WAIS-III; Wechsler, 1981), where outcome scores were number of correct repetitions for forward and backward digit span. Pattern comparison (Salthouse and Babcock, 1991) and digit cancellation (Zazzo, 1974) were used to assess *processing speed*. Thirty pattern combinations needed to be judged, as fast as possible, to be same or different within 30 s, and the outcome was average number of correct responses across two trials. For digit cancellation, a sheet with 11 rows of random digits was presented, and participants were asked to cross out the target digit (4) as fast as possible. The outcome score was number of correct responses within 30 s. *Attention and executive functioning* were assessed by the Trail Making Test (TMT; Lezak, 2004). TMT-A (attention) and TMT-B (executive functioning) each consisted of 13 circles with the same distance between them. For TMT-A, participants were required to connect encircled digits in numerical order (1, 2, 3, etc.). In TMT-B, circles included both digits and letters, and the participants’ task was to connect these in alternating order (1-A, 2-B, 3-C, etc.). One careless connection (>2 mm) was allowed, and the first mistake was corrected by the test leader without resulting in a lower score. The outcome for this task was completion times for participants with 12 correct connections. *Verbal fluency* (Lezak, 2004) required oral generation of as many words as possible beginning with a target letter (F and A; letter fluency), and generation of words belonging to a specific category (occupations and animals; category fluency). Each trial lasted 60 s. Outcomes were the average number of words generated across the two subtests for letter and category fluency, respectively. A list of 16 unrelated nouns (e.g., carrot, ring, fork) was used to assess *episodic memory* (free recall and recognition; Laukka et al., 2013). The outcome score for free recall was number of correctly recalled nouns within 2 min. Immediately following free recall, a recognition task was administered. Here, 32 nouns were presented (16 targets, 16 distracters), and participants were asked to judge whether or not a word had been presented earlier (yes–no). The outcome score for recognition was number of hits minus number of false alarms. *Semantic memory* was assessed with tests of general knowledge and vocabulary. Ten general knowledge questions (e.g., When did Aristotle live?) with two response alternatives was administered (Dahl et al., 2009), where the outcome score was number of correct answers. The vocabulary task (SRB:1; Dureman, 1960; Nilsson et al., 1997) consisted of 30 words, each presented along with five alternatives. Participants’ task was to choose the correct alternative. The outcome score was number of correctly selected synonyms within 7 min. A 10-item, simplified version (Vandenberg and Kuse, 1971; Rehnman and Herlitz, 2006) of the Shepherd–Metzler mental rotation test was used to assess *spatial ability*. A target figure plus three rotated figures were presented, and participants’ task was to decide which of the three rotated figures matched the target figure within the 45 s time limit. The outcome score was number of correctly selected figures.

### Statistical Analyses

ANOVAs and χ^2^ tests were used to assess group differences in background characteristics. Cognitive performance differences were assessed with one-way ANCOVAs (five groups), using age and gender as covariates. Regression analyses were used to examine contributions from clinical and health indicators to group differences in cognitive performance.

## Results

### Background Characteristics

Background and health characteristics across the five groups are reported in **Table 1**. Significant group differences were found for age: DNH was older than all other groups, *ps* < 0.001; DSRH was older than the remitted group, *p* < 0.01; Montgomery and Åsberg Depression Rating Scale (MADRS; Montgomery and Asberg, 1979): HC and the remitted group scored lower than all depressed groups, *ps* < 0.05; DIPH had a higher score compared to the other depressed group, *ps* < 0.01; Mini Mental State Examination (MMSE; Folstein et al., 1975); DNH was outperformed by all groups, *ps* < 0.01, except DIPH, and DIPH was further outperformed by HC and the remitted group, *p* < 0.01; prodromal dementia: DNH had a higher proportion of preclinical cases compared to all groups, *ps* < 0.05; heart disease burden: DNH had a higher burden compared to all groups, *p* < 0.05, except DIPH; physical inactivity: HC was less physically inactive relative to all depressed groups, *ps* < 0.001, but not relative to the remitted group; DIPH was more physically inactive than all other groups, *ps* < 0.05; pharmacological therapy: DNH was less medicated compared to the other groups, *ps* < 0.01; DSRH was less medicated compared to DIPH.

Clinical characteristics for groups with a psychiatric inpatient history are also reported in **Table 1**. Differences were observed for all variables (*ps* < 0.05), except age at fist admission, (i.e., both groups had an on-set before age 60). The depressed group with an inpatient history was older at last admission, had a longer psychiatric period, had fewer years since last admission, and had more admissions and inpatient days compared to the remitted group.

### Group Differences in Cognitive Performance

Reliable main effects of group were observed for pattern recognition (*F*_4,224_ = 3.05, *p* < 0.05, η^2^ = 0.052), digit cancellation (*F*_4,225_ = 3.95, *p* < 0.01, η^2^ = 0.066), TMT-A (*F*_4,224_ = 2.53, *p* < 0.05, η^2^ = 0.043), TMT-B (*F*_4,188_ = 4.04, *p* < 0.01, η^2^ = 0.079), letter fluency (*F*_4,243_ = 4.76, *p* < 0.001, η^2^ = 0.073), and category fluency (*F*_4,242_ = 3.64, *p* < 0.01, η^2^ = 0.057). See **Table 2** for raw scores.

**Table 2:** Raw scores of cognitive performance across depression status and psychiatric history.

Cognitive tests	Healthy controls (HC) *n* = 96		Depressed no history (DNH), *n* = 49		Depressed self-reported unipolar depression (DSRH), *n* = 52		Depressed inpatient history mixed (DIPH), *n* = 20		Remitted inpatient history unipolar depression (RIPH), *n* = 38	
	*M*	(SD)	*M*	(SD)	*M*	(SD)	*M*	(SD)	*M*	(SD)
**Processing speed**										
Pattern comparison	14.52	(4.38)	10.86	(3.79)	12.78	(4.02)	12.72	(2.66)	14.24	(4.28)
Digit cancellation	17.72	(4.21)	13.98	(4.03)	17.12	(3.07)	15.06	(4.03)	17.66	(4.38)
**Short term memory**										
Digit Span forward	6.99	(1.87)	6.44	(1.67)	7.02	(2.03)	6.58	(1.47)	6.53	(1.80)
Digit Span backward	5.85	(2.04)	5.13	(1.98)	6.14	(2.23)	5.21	(1.62)	5.80	(2.04)
**Attention and executive function**										
TMT-A, time (seconds)	14.27	(6.57)	21.81	(15.45)	16.09	(7.22)	17.82	(8.23)	14.25	(5.40)
TMT-B, time (seconds)	27.43	(12.16)	44.48	(30.93)	35.18	(17.03)	42.15	(22.26)	28.19	(17.30)
**Verbal fluency**										
Letter fluency	13.93	(5.12)	10.67	(4.86)	13.63	(5.65)	10.03	(4.08)	13.88	(4.20)
Category fluency	18.89	(5.86)	14.72	(5.38)	16.66	(4.98)	14.83	(4.17)	19.12	(5.81)
**Episodic memory**										
Free recall	7.43	(2.71)	5.89	(2.03)	6.29	(2.48)	6.18	(1.98)	6.95	(2.48)
Recognition	11.59	(3.09)	11.20	(3.48)	10.38	(3.28)	11.35	(2.47)	11.40	(2.64)
**Semantic memory**										
Vocabulary	22.79	(5.34)	19.84	(6.24)	21.81	(5.84)	23.29	(3.65)	22.39	(6.90)
General knowledge	6.90	(1.77)	6.83	(1.34)	6.94	(1.70)	7.06	(1.21)	7.03	(1.72)
**Spatial ability**										
Mental rotation	6.37	(1.78)	5.51	(1.57)	5.84	(1.96)	5.56	(1.46)	6.08	(1.68)

*M, mean; SD, standard deviation*.

Follow-up analyses revealed that HC outperformed DIPH and DNH for all significant outcomes (*ps* < 0.05), except for TMT-A (DIPH), and also outperformed DSRH for pattern comparison (*ps* < 0.05). DIPH was further outperformed by DSRH and the remitted group on digit cancellation, letter and category fluency, and on TMT-B by the remitted group (*ps* < 0.05). DNH was outperformed by DSRH on digit cancellation and letter fluency, and by the remitted group on letter fluency (*p* < 0.05).

A trend level group effect was observed for episodic free recall (*p* = 0.074), whereas no deficits were observed for semantic memory or spatial ability.

Calculations of effect sizes (Cohen’s *d;* Cohen, 1992) revealed medium to large performance advantages of HC and depressed with self-reported history, and medium advantages for the remitted group, see **Table 3**.

**Table 3:** Effect sizes (Cohen’s *d*) for significant cognitive group differences.

	Pattern comparison	Digit cancellation	TMT-A	TMT-B	Letter fluency	Category fluency
HC > DNH	1.13	1.00	0.61	0.59	0.66	0.80
HC > DSRH	0.50	n.s.	n.s.	n.s.	n.s.	n.s.
HC > DIPH	0.63	0.50	n.s.	0.85	0.66	0.88
DSRH > DIPH	n.s.	0.57	n.s.	n.s.	0.66	0.50
DSRH > DNH	n.s.	1.13	n.s.	n.s.	0.66	n.s
RIPH > DIPH	n.s.	0.50	n.s.	0.71	0.75	1.10
RIPH > DNH	n.s.	n.s.	n.s.	n.s.	0.75	n.s.

*n.s., non-significant. HC, Healthy controls; DNH, depressed, no history; DSRH, depressed, self-reported history, unipolar; DIPH, depressed, inpatient history, mixed; RIPH: remitted, psychiatric inpatient history unipolar*.

### Effects of Background Characteristics on Group Differences in Cognitive Performance

As complementary analyses, a series of regression analyses were run to identify contributions of different characteristics to group differences in cognitive performance. These analyses focused on cognitive tasks where reliable group differences were observed in conjunction with reliable group differences in background, health, or clinical characteristics (see above).

Heart disease burden (coronary heart disease, heart failure, and atrial fibrillation) had significant effects for all cognitive outcomes on the performance differences between HC and DNH (*ps* < 0.01), except for TMT-A, so that group differences were attenuated when this variable was included. Change in beta coefficients, calculated as 100∗(beta1–beta2)/beta1, ranged from 23.3% (category fluency) to 32.9% (pattern comparison). For TMT-A, prodromal dementia modulated the performance difference between HC and DNH (*p* < 0.01) with a change in beta coefficient of 38.6%. Heart disease burden continued to modulate performance differences between DSRH and DNH on digit cancellation (*p* < 0.01) and letter fluency (*ps* < 0.05) with change in beta coefficients of 21.9, and 12.3%, respectively.

Physical inactivity had significant effects on all outcomes on the performance differences between HC and DIPH (*ps* < 0.05), with change in beta coefficients ranging from 31.3% (TMT-B) to 76.7% (pattern comparison). Physical inactivity also modulated the performance difference between the remitted and DIPH for letter fluency (*p* < 0.05), with a change in beta coefficient of 39.3%.

Number of cumulative inpatient days had significant effects on performance differences between the remitted group and DIPH for digit cancellation (*p* < 0.05) and TMT-B (*p* < 0.001). Change in beta coefficients were 52.4 and 84.9%, respectively. Age at last admission modulated the performance difference between the remitted group and DIPH for digit cancellation (*p* < 0.05), with a 33.9% change in beta coefficient.

Medication status and years since last inpatient admission did not contribute to the observed group differences in cognitive performance.

## Discussion

The main findings of this study were that; (a) among currently depressed persons, those with a psychiatric inpatient history, and late onset performed at the lowest levels, (b) remitted unipolar depression exhibited no cognitive deficits.

### Cognitive Performance in Current Depression

In line with previous research, currently depressed persons exhibited deficits in processing speed, attention, executive functions, and verbal fluency (Beats et al., 1996; Lockwood et al., 2002; Butters et al., 2004; Ganguli et al., 2009; Thomas et al., 2009; Köhler et al., 2010).

Depression is linked to neurobiological alterations (e.g., at functional, structural, and neurochemical levels), especially in the prefrontal cortex, and the temporal lobes (for reviews see Drevets, 2000; Lorenzetti et al., 2009). In addition, the prefrontal cortex has been shown to be especially sensitive to corticosteroids (i.e., stress; Lupien et al., 1999; Young et al., 1999). This is consistent with the observed depression-related deficits in processing speed, attention, executive functions, and verbal fluency, all of which draw on the prefrontal cortex.

Depressed persons with recurrent mixed psychiatric inpatient history consistently performed at a low level, indicating that psychiatric inpatient history is related to poorer cognitive performance in old-age depression. Recurrent depression has been associated with exacerbated cognitive deficits, a possible reason being that depressive symptoms worsens with each new episode (Takami et al., 2007; Robinson and Sahakian, 2008).

Neurobiologically, this may be reflected in prolonged stress-reactivity on the hypothalamic-pituitary-adrenal (HPA) axis, causing damaging levels of corticosteroids, and cortisol (see Pariante and Lightman, 2008). In conjunction with the presence of psychiatric co-morbidity, which has been shown to be a predictor of cognitive deficits in depression (Baune et al., 2009), and the vulnerabilities of an aging brain (Bäckman et al., 2000; Raz et al., 2005), it is therefore not unexpected that DIPH would perform at a low cognitive level. However, note that psychiatric inpatient history *per se*, without a current depressive episode (i.e., the remitted group), did not affect cognitive performance. Thus, cognitive deficits were only observed in combination with current depression.

Although lower cognitive performance in DIPH was an expected finding, performance of persons with late-onset current depression (DNH) was at a similar low level. Whereas cumulative psychiatric illness may explain the poor cognitive performance of DIPH, heart disease burden was shown to significantly contribute to performance differences for DNH for all significant cognitive outcomes. An exception was TMT-A, where prodromal dementia modulated the performance difference. Co-morbidity of cardiovascular disease and depression is highly frequent, and also well known to have a bidirectional association (Barth et al., 2004; Halaris, 2013). Further, heart disease is also known to be associated with cognitive deficits (Pressler et al., 2010; Dardiotis et al., 2012). Taken together, high frequencies of vascular disease (Alexopoulos, 1997), and prodromal dementia, which is well known to cause broad cognitive deficits (Bäckman et al., 2005), may have resulted in a comparable negative impact on cognition as recurrent psychiatric episodes requiring inpatient care.

### Cognitive Performance in Remitted Unipolar Depression

The remitted group performed at the same cognitive level as HC. The lack of cognitive deficits in remitted recurrent unipolar depression is an interesting finding, as past research suggests otherwise (Beats et al., 1996; Nebes et al., 2000; Portella et al., 2003; Yuan et al., 2008; Hasselbalch et al., 2011, 2013; Bora et al., 2013). A recent review on cognitive functioning in remitted depression (Douglas and Porter, 2009) suggests that deficits in attention and executive functions may be trait markers of depression, whereas deficits in other cognitive domains may be state-related. However, heterogeneity of definitions and durations of remission make previous results difficult to interpret. For example, remission definitions varied from MADRS scores of 50% improvement, to a score less than 10, 9, or 8. Durations typically ranged from 1 to 6 months, or were not specified (Beats et al., 1996; Beblo et al., 1999; O’Brien et al., 2004; Lee et al., 2007). Studies on cognitive performance and long-term remission in depression are scarce, but one study with a remission duration of 6 years reported a remaining deficit in global cognitive functioning (Hasselbalch et al., 2013). On the other hand, a study with a follow-up time of 10 years reported normalization of effortful information processing (Hammar and Årdal, 2012).

We report a complete lack of cognitive deficits in remitted depression in old-age. Conceivably, a key factor here is the extended time since the last admission (m = 15.6 years). Furthermore, this remitted group had a MADRS score of 2.6, and thus few residual depressive symptoms. These results, along with those of prior research, suggest that cognitive deficits in unipolar depression may be more state- than trait-related, and take considerably longer time to remit in comparison to depressive symptoms.

### Physical Inactivity, Cumulative Inpatient Days, Age at Last Admission

In analyzing factors contributing to cognitive group differences, we found that physical inactivity (all cognitive outcomes), cumulative inpatient days (processing speed, executive functions), and older age at last admission (processing speed), modulated performance differences between DIPH and the HC as well as with the remitted group. Thus, these factors may partly explain the poor cognitive performance observed for DIPH. As cumulative illness burden is associated with relapse (APA, 2014), and physical inactivity is associated with old-age depression (Wassink-Vossen et al., 2014), these factors may have contributed to relapse for DIPH, and also to the exacerbated cognitive deficits observed in this group (Takami et al., 2007; Robinson and Sahakian, 2008).

### Limitations

Matching older SNAC-K participants to the IPR provided an opportunity to target all primary psychiatric inpatient diagnoses dating 35 years back in time. However, given the time span, with changes from ICD-8 to -9- and -10, and differences in clinical settings – the overall positive predictive value (PPV) of diagnoses in the IPR is estimated to be 85–95%, and 3.1% of psychiatric primary diagnoses are estimated to be missing (Ludvigsson et al., 2011). Also, treatment information (e.g., pharmacological, ECT) received during admissions was lacking. Because findings from this cross-sectional study suggest that cognitive deficits in unipolar depression may be more state than trait related, longitudinal research on long-term remission, and cognitive performance in aging is warranted.

### Implications

Relapse rates in psychiatric disorders remain high (APA, 2014). Cognitive symptoms are associated with higher relapse rates (Dunkin et al., 2000; Jaeger et al., 2006) and reduced quality of life (Shimuzu et al., 2013). As shown in this study, they may also take long time to remit. Therefore, combined profiles of psychiatric history, cognitive performance and health behaviors may provide important clues to individualized treatment. This is especially so as 30–60% of depressed persons may be classified with treatment-resistant depression (Fava, 2003). For example, a depressed older person may benefit from aerobic exercise not only in terms of symptom relief (Greer and Trivedi, 2009), but also with regard to executive functioning (Colcombe and Kramer, 2003).

## Conclusion

Currently depressed persons exhibited deficits in processing speed, attention, executive functions, and verbal fluency, and those with a psychiatric inpatient history and late-onset depression performed at the lowest levels. Another important finding is that cognitive residuals in unipolar depression may largely be a function of time spent in remission; as we observed no cognitive deficits in the remitted group.

## Author Contributions

AP, conception of the study, execution of statistical analyses, manuscript first draft. EL, conception of the study, review and critique of the manuscript, supervised all stages of the study. AA, made all depression diagnoses, review and critique of the manuscript. LB, wrote the protocol for the cognitive data collection in SNAC-K, conception of the study, review and critique of the manuscript, supervised all stages of the study.

## Conflict of Interest Statement

The authors declare that the research was conducted in the absence of any commercial or financial relationships that could be construed as a potential conflict of interest.
